# Central Retinal Vein Occlusion in a Young Healthy Man Following Strenuous Exercise: Multimodal Imaging and Treatment Response

**DOI:** 10.7759/cureus.108935

**Published:** 2026-05-15

**Authors:** Parul Jain, Avinash Pradhan, Pushkar Rangari, Paromita Dutta

**Affiliations:** 1 Ophthalmology, Maulana Azad Medical College, New Delhi, IND; 2 Cornea, Maulana Azad Medical College, New Delhi, IND

**Keywords:** anti-vegf therapy, central retinal vein occlusion, cystoid macular edema, idiopathic crvo, optical coherence tomography, retinal vascular occlusion, strenuous exercise, valsalva maneuver, weightlifting, young adult

## Abstract

Central retinal vein occlusion (CRVO) is uncommon in young, otherwise healthy individuals and is often associated with underlying systemic or hematological risk factors. We report a case of a 23-year-old healthy man who presented with sudden painless diminution of vision in the right eye shortly after engaging in strenuous physical exercise involving heavy weightlifting. Best-corrected visual acuity at presentation was 6/18. Fundus examination revealed optic disc edema, dilated and tortuous retinal veins, and widespread intraretinal hemorrhages consistent with non-ischemic CRVO. Optical coherence tomography (OCT) demonstrated significant cystoid macular edema with a central macular thickness of 823 µm and associated subretinal fluid.

Comprehensive systemic evaluation, including hematological, metabolic, coagulation, autoimmune, cardiac, and carotid workup, was unremarkable. The patient was treated with intravitreal anti-vascular endothelial growth factor (anti-VEGF) therapy, resulting in marked anatomical improvement and visual recovery to 6/9 on follow-up.

Although CRVO in young patients is rare and often idiopathic, intense physical exertion may act as a potential precipitating factor through transient hemodynamic changes such as increased venous pressure, Valsalva-like mechanisms, and hemoconcentration. This case highlights the importance of considering recent physical stressors in the clinical history and demonstrates a favorable treatment response with anti-VEGF therapy.

## Introduction

Central retinal vein occlusion (CRVO) is one of the most common retinal vascular disorders and a significant cause of visual impairment worldwide. It is classically seen in older individuals and is strongly associated with systemic risk factors such as hypertension, diabetes mellitus, hyperlipidemia, and hypercoagulable states. The underlying pathogenesis involves thrombotic occlusion of the central retinal vein, typically at the level of the lamina cribrosa, leading to venous congestion, retinal hemorrhages, and macular edema, which is the principal cause of visual loss [1-3].

In contrast, CRVO in young adults is relatively uncommon and often presents a diagnostic challenge. Many patients in this age group lack conventional systemic risk factors, and despite extensive evaluation, the condition is frequently categorized as idiopathic. Studies focusing on young CRVO populations have demonstrated a heterogeneous profile, with a significant proportion of patients showing no identifiable etiology even after detailed systemic and ocular workup [2-4]. This underscores the need to explore additional or transient contributing factors in such cases.

Strenuous physical activity has been proposed as one such potential precipitating factor. Activities involving heavy weightlifting are known to induce Valsalva-like maneuvers, resulting in transient increases in intrathoracic and venous pressure. These hemodynamic changes may impair retinal venous outflow and promote venous stasis. Rouhani et al. reported cases of CRVO in otherwise healthy individuals following intense exercise, suggesting a possible association mediated by transient vascular changes [1]. Additionally, exercise-related dehydration and hemoconcentration may further increase blood viscosity, potentially contributing to vascular occlusion, although these mechanisms remain theoretical. Non-ischemic CRVO generally carries a more favorable visual prognosis compared with ischemic variants, although both forms require careful systemic and ocular evaluation. Recognition of uncommon precipitating factors in young, otherwise healthy individuals may aid in early diagnosis and appropriate management.

Despite these observations, the relationship between strenuous exercise and CRVO is not well established, and current evidence is limited to isolated reports. A definitive causal link has not been proven, and such associations should be interpreted with caution. In this context, we report a case of non-ischemic CRVO in a young healthy man presenting in temporal association with recent strenuous exercise, with no identifiable systemic risk factors on detailed evaluation. This case highlights the importance of thorough clinical assessment and careful consideration of transient physiological stressors in young patients presenting with retinal vascular occlusion.

## Case presentation

A 23-year-old man presented with a diminution of vision in the right eye. The patient developed sudden painless diminution of vision shortly after a session of strenuous heavy weightlifting exercise. His best-corrected visual acuity (BCVA) was 6/18 in the right eye and 6/6 in the left eye. He reported recent strenuous gym activity involving heavy weightlifting, along with regular intake of protein supplementation before the onset of symptoms. There was no history of anabolic steroid use, testosterone intake, stimulant use, or recreational drug use.

He had undergone uneventful laser-assisted in situ keratomileusis (LASIK) for myopia 10 months earlier and had received a Covishield COVID-19 booster dose four years ago; both were considered remote and unlikely to be contributory.

Anterior segment examination and intraocular pressure were within normal limits in both eyes.

**Figure 1:**
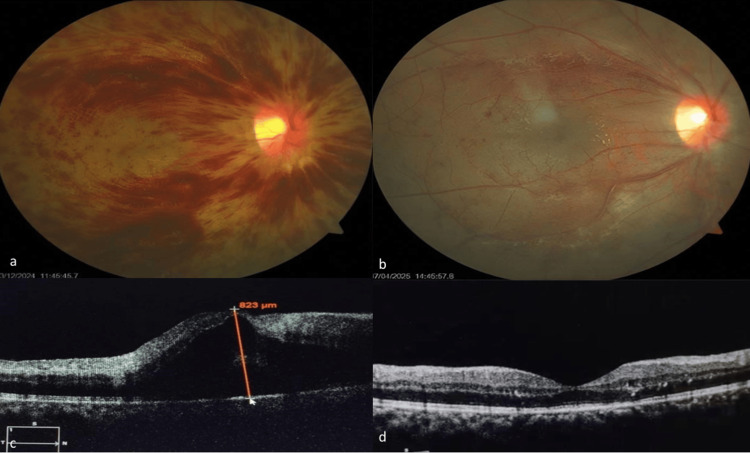
Multimodal imaging of central retinal vein occlusion in a young healthy adult following strenuous exercise. (a) Baseline color fundus image of the right eye showing optic disc edema, dilated and tortuous retinal veins, and widespread intraretinal hemorrhages. (b) Follow-up fundus image demonstrating a significant resolution of retinal hemorrhages and reduction in venous congestion following intravitreal anti-VEGF therapy. (c) Baseline optical coherence tomography (OCT) macula showing marked cystoid macular edema with multiple intraretinal cystic spaces and increased central macular thickness of 823 µm. (d) Follow-up OCT macula demonstrating significant reduction in intraretinal fluid with restoration of foveal contour and improved retinal architecture after treatment. VEGF: vascular endothelial growth factor

Fundus examination of the right eye revealed optic disc edema, dilated and tortuous retinal veins, and widespread intraretinal hemorrhages with macular edema, consistent with non-ischemic CRVO (Figure 1a). The left eye was unremarkable.

Optical coherence tomography (OCT) of the right eye demonstrated cystoid macular edema with a central macular thickness of 823 µm and associated subretinal fluid (Figure 1c).

Comprehensive systemic evaluation, including hematological parameters, blood glucose, lipid profile, coagulation profile, autoimmune markers, cardiac evaluation, and carotid Doppler, was within normal limits, and no underlying systemic risk factor was identified.

The patient received intravitreal anti-vascular endothelial growth factor (intravitreal bevacizumab) therapy. At four-week follow-up, BCVA improved to 6/9, with a significant reduction in macular edema to 275 µm on OCT (Figure 1d) and clinical resolution of retinal hemorrhages (Figure 1b).

## Discussion

CRVO in young individuals without systemic comorbidities is uncommon and frequently categorized as idiopathic. Studies have demonstrated that a significant proportion of young patients lack identifiable risk factors even after extensive evaluation [2-4]. A comprehensive differential evaluation excluding inflammatory, thrombotic, autoimmune, and systemic vascular causes is essential in young patients presenting with CRVO.

In recent years, attention has been drawn to transient physiological factors that may contribute to retinal vascular events in otherwise healthy individuals. Strenuous physical activity, particularly heavy weightlifting, is associated with Valsalva-like maneuvers that can result in abrupt increases in intrathoracic pressure and venous pressure. These changes may impair retinal venous drainage and lead to transient venous stasis, potentially predisposing to vascular occlusion.

Rouhani et al. reported similar cases of CRVO occurring after intense exercise in healthy individuals, supporting the hypothesis that transient hemodynamic alterations may play a role [1]. Additionally, dehydration and hemoconcentration associated with vigorous exercise may increase blood viscosity and further contribute to vascular compromise.

In the present case, the absence of systemic risk factors and the temporal association with strenuous exercise suggest a possible contributory role of exercise-induced hemodynamic changes. However, given the limited evidence and lack of direct causative proof, this association should be interpreted cautiously. The patient demonstrated a favorable response to intravitreal anti-VEGF therapy, with both anatomical and visual improvement, consistent with the expected course of non-ischemic CRVO.

## Conclusions

This case highlights the occurrence of non-ischemic CRVO in a young, otherwise healthy individual in temporal association with strenuous physical activity. While exercise-related hemodynamic changes may be a potential contributing factor, a definitive causal relationship cannot be established. Thorough systemic evaluation remains essential in young patients with CRVO, and early treatment with anti-VEGF therapy can lead to favorable visual outcomes. This case highlights the importance of considering uncommon or transient precipitating factors in young patients presenting with retinal vascular occlusion despite the absence of conventional systemic risk factors. However, the association between strenuous exercise and CRVO remains speculative, and conclusions are limited by the single-case nature of the report.
